# Some Properties of Chemical Cross-Linking Biohydrogel from Starch and Chitosan

**DOI:** 10.1155/2019/1542128

**Published:** 2019-03-05

**Authors:** Titi Candra Sunarti, M. Irsan Febrian, Eka Ruriani, Indah Yuliasih

**Affiliations:** Department of Agroindustrial Technology, Bogor Agricultural University, Kampus IPB Dramaga, PO Box 220, Bogor, 16680, Indonesia

## Abstract

Chemical cross-linking was developed to prepare starch and chitosan-based hydrogels. First, the precursor of starch was synthesized through the reaction of carboxymethylation with sodium monochloroacetate, and then chitosan was grafted by using methacrylic acid as cross-linker. In this research, sago and cassava starches were used and mixed with chitosan, and the effect of methacrylic acid concentration was investigated to determine the grafting parameters and hydrogel characteristics. Compared to native starch and carboxymethylated starch, hydrogels from both starches have high ability to swell and high capacity to absorb water and oil. The highest grafting yield, grafting efficiency, and monomer conversion were achieved by experiment using 0.550 g of methacrylic acid per g of CMS-chitosan mixture. These hydrogels have a good potency as biodegradable absorbents for pharmaceutical and industrial application.

## 1. Introduction

The term hydrogel describes three-dimensional network structures obtained from a class of synthetic and/or natural polymers which can absorb and retain significant amount of water [1]. The hydrogel structure is created by the hydrophilic groups or domains present in a polymeric network upon the hydration in an aqueous environment. Hydrogel has many applications as diapers, additives in agriculture, contact lenses, and matrices in drug delivery system. The swelling characteristic of hydrogel is influenced by the chemical structure of hydrogel polymers, containing hydrophilic functional groups, and will have better swelling capability compared to hydrogel containing hydrophobic group [2].

The classification of hydrogel is based on (i) the source, i.e., natural, synthetic, and hybrid hydrogels (consisting of natural and synthetic molecules); (ii) the type of the cross-linking; it can take place as covalent linking and physical linking gels (iii); the nature of the network categorized as homopolymer, copolymer, interpenetration, or double chain; (iv) its physical structure, classified into homogeneous (optical transparent), micropore, and macropore; (e) the nature of the organism, differentiated into biodegradable and nondegradable hydrogels [3].

Native starch has limited application as active compound matrix since it has multihydroxyl structure and hydrophilic groups, which will cause quick release of active compounds before the target [4]. Modification will improve the starch crystallinity and hydrophobicity and amphiphilic properties with hydrophilic and hydrophobic sides or have helical cavity which can form active compound inclusion complexes.

Some researchers have reported the production of starch-based hydrogel. Biodegradable hydrogel based on hydroxy methacrylate starch shows the sustained release properties for the* in vitro *FITC-anti-human antibodies and* in vivo *IgG-FITC [5]. Superabsorbent hydrogel is synthesized from copolymerization cross-linking of modified vinyl-starch and acrylic acid induced by UV light [6, 7].* In situ* hydrogel constructed by starch-based nanoparticle and polyvinylamine via a Schiff base reaction is used to test the controlled release of doxorubicin [7]. Synthesis of sodium carboxymethyl starch from high amylose starch for the application of controlled release matrix for oral medications has been reported in [8]. Starch-based nanosphere is cross-linking agent to make polyacrylamide hydrogel with high mechanical resistance [9].

Chitosan is a natural, hydrophilic, nontoxic, biocompatible, and biodegradable polysaccharide suitable for application in pharmaceutical technology. Chitosan is soluble at acidic pH, forming gel [10]. Solubility of chitosan in aqueous solutions is attained via protonation of its amine groups in acidic environment. Chitosan exists in different molecular weights and degrees of acetylation. The intermolecular forces between polysaccharide chains of chitosan are hydrogen, hydrophobic, and ionic interactions. But these interactions are influenced by molecular weight and ionic strength. Cross-linking of chitosan polymers is necessary to improve chitosan properties such as stability and durability for the aim of drug delivery [11]. In this research, sago and cassava starches were modified through cross-linking to form swellable and nontoxic carboxymethyl starch, and then to improve its compatibility, the mixture of carboxymethyl starch and chitosan was copolymerized with methacrylic acid.

## 2. Materials and Methods

### 2.1. Materials

Native sago and tapioca starches were purchased from local starch industries in Bogor, while chitosan was kindly supplied from Department of Marine Products, Bogor Agricultural University, with medium molecular weight and relatively high degree of deacetylation (93.8%).

#### 2.1.1. Preparation of Carboxymethyl Starch

All starches were dried and pulverized to pass the 80 mesh screen, and then analysed for amylose and moisture content for determining the anhydroglucose unit (AGU) in modifying of starch. Carboxymethylation of the starches was conducted by using of [12] method with slight modification. Starch was suspended in isopropanol with the ratio of starch weight to propanol volume as 1:10 and added with 1.8 molar ratio of NaOH to AGU. The mixing was conducted on 40°C continuous stirrer with 250 rpm of speed for 15 min and then continued for 3 hr after the addition of 1.3 molar ratio of sodium monochloroacetate (SMCA) to AGU. The mixture was maintained the pH ranging from 5.5 to 6.5 with 50% of HCl. Then the mixture was precipitated and washed several times by using 85% of ethanol and filtered until the filtrate gave negative response against AgNO_3_ solution. Finally, the pure product was oven-dried at 50°C. The characteristics of CMS were determined as degree of substitution (DS) according to ISO 11216-1998 method [13], solubility and swelling power at 70°C [14], and water and oil absorption capacities [15].

#### 2.1.2. Synthesis of Biohydrogel

Production of biohydrogel was conducted according to [12] with modification. One part of the polymer mixtures, consisting of 12 parts of CMS and a part of chitosan, was diluted in 20 parts of 1% of formic acid. The mixing was conducted on 40°C with continuous stirring (250 rpm) under nitrogen atmospheric condition. Copolymerization was initiated by ceric ammonium nitrate (CAN) as an initiator and methacrylic acid as a cross-linking agent. The 0.005 M of CAN diluted in 1 M of HNO_3_ was added to the homogenous mixture and then followed by addition of different molar ratio of methacrylic acid. The reaction was conducted for one hour, and then the product was neutralized with 1.25 N of NaOH solution, precipitated by methanol, filtered, and oven-dried on 70°C. The crude product was purified, rewashed with ethanol several times to dilute the excess of methacrylic acid, and then oven-dried. The presence of certain functional group in molecule was detected by Fourier transform infrared spectroscopy.

### 2.2. Determination of Grafting Parameters

Grafting parameters, grafting yield (%GY) (1), grafting efficiency (%GE) (2), and monomer conversion (%MC) (3), were determined according to [16], as follows:

(1) %GY=W2−W1W0×100%

(2) %GE=W2−W0W1−W0×100%

(3) %MC=W1−W0Wn×100%

where W_0_ is the weight of the mixture of CMS and chitosan, W_1_ is the weight of crude product, W_2_ is the weight of pure product, and W_n_ is the weight of methacrylic acid.

### 2.3. Characterization of Biohydrogel Properties

Characterization of hydrogel solubility and swelling properties was conducted according to Japanese Industrial Standard-JIS K7223 [13]. Water and oil absorption capacities were determined according to [16] methods with slight modification.

## 3. Results and Discussion

### 3.1. Synthesis and Characteristics of Sodium Carboxymethyl Starch

Carboxymethyl starch is anionic starch synthesized through etherification on hydroxyl group of glucose [17]. This modification aims to insert the hydrophilic group in the AGU of starch in order to stabilize it in the aqueous media and prevent its retrogradation at low temperature. Carboxymethyl starch is produced by using two steps. First, starch solubilisation with alcohol-alkaline treatment and then continuing to the reaction of starch and sodium monochloroacetate (SMCA) [12]. When starch molecules were placed in a strong alkaline solution, the hydroxyl groups (-OH) of starch molecules were activated and transformed into the more reactive alkoxide form (StO^−^) (4) [12]. Alkaline condition opens up the starch granule structure, resulting in breaking the intermolecular hydrogen bonds there by enhancing the water solubility [18]. Protons of the -OH group were dissociated and left negative charges on starch molecules. The repulsion between negative charges resulted in swelling of starch granules [19]. These properties will induce the etherification process to distribute the SMCA through amorphous starch and to form sodium carboxymethyl starch (5) [20].

(4) $$\begin{array}{c} St\text{-}OH+NaOH⟷St\text{-}ONa+H_{2}O \end{array}$$

(5) $$\begin{array}{c} St\text{-}ONa+Cl\text{-}{CH}_{2}\text{-}CO\text{-}ONa⟷ \end{array}$$

The properties of CMS are mainly determined by the degree of substitution (DS) and the average number of carboxymethyl groups per anhydroglucose unit (AGU) [21]. In this research the DS of CMS is 0.60 and 0.53 for cassava and sago starch, respectively. Carboxymethylation can improve the swelling and solubility of native cassava and sago starches (Table 1). Amylose content in starch contributed to the solubility [22]. In this research, sago starch showed slightly higher solubility compared to cassava starch, since sago and cassava starches contained 23.05% and 22.28% of amylose contents, respectively. Sodium carboxymethylated starch is cold water soluble starch and has high disperse distribution and excellent water absorption capacities compared to native starch.

### 3.2. Synthesis and Characteristics of Biohydrogel

Hydrogel can be synthesized by physical and chemical treatments. In this research, hydrogel composites based on CMS and chitosan cross-linked chemically. Both polymers, CMS and chitosan, are soluble polymers in acid condition, and CMS is the main backbone with ratio 12:1 to chitosan. It has been shown that C6 of starch has been partially substituted by carboxymethyl group, and C2-C3 are predominant sites for the initiation of graft copolymerization. Chitosan has been extensively used to synthesize hydrogels due to its cross-linking ability, which has been attributed to the free amine groups in its backbone [23]. Blending of starch with chitosan has increased the elasticity and flexibility in the resulting polymeric hydrogel, as its application in controlled release delivery of *α*-hydroxy acid contained in tamarind fruit pulp extract has been reported [24].

Cross-linking covered here involves grafting of monomers on the backbone of the polymers and the use of a cross-linking agent to link two polymer chains. The cross-linking of carboxymethyl starch and chitosan polymers can be achieved through the reaction of their functional groups (OH, COOH, and NH_2_) with cross-linking agent-methacrylic acid. Grafting process of methacrylic acid monomer on CMS-chitosan backbone is conducted through the formation of these radical in the functional groups.

**Figure 1:**
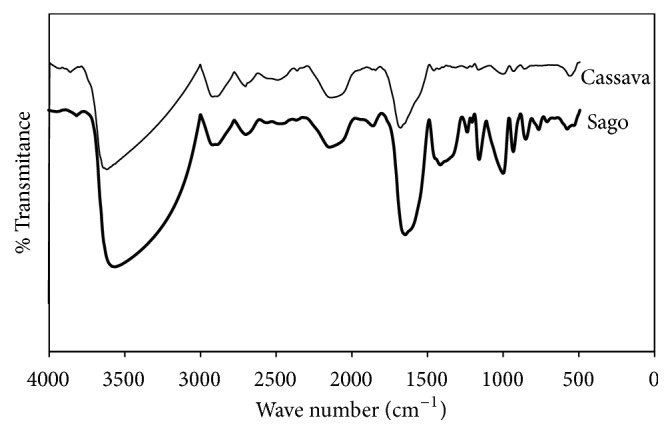
FT-IR spectra of biohydrogel from carboxymethyl starch-chitosan.

The formation of these radical groups will trigger the formation of copolymers and cross-linking between polymer backbones. Chemical free radical formation in the polymer backbone functional group can use the ceric ammonium nitrate. The success of the grafting process can be identified by the presence of certain functional group in molecule and detected by Fourier transform infrared spectroscopy. FT-IR spectra of hydrogel from each CMS are shown in Figure 1.

Substitution of carboxymethyl group on sago and cassava starch can be identified with the presence of some peaks ranging between 1300 and 860 cm^−1^, which are attributed to the stretching vibrations of C-O in C-O-C and C-O-H from glycosidic molecules. The presence of carboxymethyl group was detected and identified as the peak around 1400 cm^−1^ is attributed to the COO- unsymmetrical and around 1600 cm^−1^ to the symmetrical stretching vibrations [25]. Related to previous research, the peaks at 1597 and 1417 cm^−1^ are attributed to the COO- unsymmetrical and symmetrical stretching vibration, respectively [26].

The wide peak around 3600 cm^−1^ is attributed to the -OH, -NH, and CH stretching vibrations of carboxymethyl starch-chitosan, while [26] reported that the wide peak around 3411 cm^−1^ was attributed to the O-H stretching vibrations of CS/CMS. The presence of a new peak at 2700 cm^−1^ is attributed to the C=C stretching vibration of methacrylic acid terminus and is also the strong evidence of the grafting process of methacrylic acid on CMS-chitosan backbone. In this case, no peak was detected at 3250 cm^−1^, and it might be the evidence that methacrylic acid did not graft on primary amine group (-NH) of chitosan.

Most commonly, graft polymerization is brought about by free radical addition polymerization. The sequence of the steps with CAN (ceric ammonium nitrate) as initiator is begun by the formation of Ce^4+^ radical reacts with starch and chitosan to form a starch radical or chitosan radical in oxidation (6) as reported in [27]. The subsequent reaction with methacrylic acid results in the formation of polymethacrylic acid grafts on the starch (7) and (9) and chitosan backbone in initiation and propagation steps. But Ce^4+^ radical not only reacts with starch or chitosan but may also serve as initiator for methacrylic acid homopolymerization (8) and (10), producing ungrafted homopolymer. Clearly, reaction conditions have to be chosen in such a way as to promote grafting onto the starch and chitosan and to reduce the formation of homopolymers. This means that the grafting efficiency should be maximized.

(6) $$\begin{array}{c} St+{Ce}^{4+}⟶Complex⟶St+{Ce}^{3+}+H^{+} \end{array}$$

(7) $$\begin{array}{c} St+M⟶StM+M⟶{StM}_{1}+M_{n}⟶ \end{array}$$

(8) $$\begin{array}{c} M^{+}+M_{n}⟶M_{n+1} \end{array}$$

(9) $$\begin{array}{c} St{\left(\;M\;\right)}_{n+1}+{Ce}^{4+}⟶St{\left(\;M\;\right)}_{n+1}+{Ce}^{3+} \end{array}$$

(10) $$\begin{array}{c} M_{n+1}+{Ce}^{4+}⟶M_{n+1}+{Ce}^{3+} \end{array}$$

Grafting of methacrylic acid to CMS-chitosan backbone produced copolymer of CMS/chitosan-g-poly(methacrylic acid) and can be evaluated by grafting parameters as shown in Table 2. Grafting yield (%GY) is defined as comparison of the weight of methacrylic acid to the initial materials, grafting efficiency (%GE) is counted as the amount of methacrylic acid which is grafted to the polymer backbone, and monomer conversion (%MC) is measured as the amount of grafted methacrylic acid to the added one. The concentration of methacrylic acid is significantly influenced by the grafting yield and monomer conversion for CMS from both sago and cassava starches. Highest grafting yield (46-47%) and monomer conversion (98-99%) is produced from the utilization of 0.550 g of methacrylic acid per g of CMS-chitosan. The increasing of methacrylic acid concentration will reduce the medium pH which causes the degradation of initiator reagent. The absence of initiator reagent will stop the radicalization process of functional group and affect the unpolymerized methacrylic acid.

High %GE value is influenced by the utilization of proper initiator. In this research, ceric ammonium nitrate (CAN) was applied, as suggested by previous research [28, 29]. CAN is generally used as initiator in grafting process since it needs low energy for activation and short time for radical forming, and it has high grafting efficiency compared to other initiators. Monomer conversion (%MC) plays an important role in biohydrogel production, since it showed the effectiveness of polymerization reaction. But the %GY and %GE can be used for determining the amount of monomer grafted to the polymer backbone and the amount of formed homopolymer.

Existence of polar functional groups such as carboxylic acid is needed not only for bioadhesive properties but also for pH-sensitive properties of polymer, because the increase of methacrylic acid content in the hydrogels provides more hydrogen bonds at low pH and more electrostatic repulsion at high pH [26].

The term hydrogel describes three-dimensional network structures obtained from a class of synthetic and/or natural polymers which can absorb and retain significant amount of water [1]. The hydrogel structure is created by the hydrophilic groups or domains present in a polymeric network upon the hydration in an aqueous environment. The swelling behaviour of the hydrogels was dependent on the content of methacrylic acid groups and caused a decrease in gel swelling for methacrylic concentration more than 0.550 or an increase in gel swelling in the lower range, as shown in Table 3.

The water binding or absorption capacity and permeability are the most important characteristic features of a hydrogel. The polar hydrophilic groups are the first to be hydrated upon contact with water which leads to the formation of primary bound water. Secondary-bound water is formed as a result of the network swells and exposes the hydrophobic groups which are also capable of interacting with the water molecules. The network will absorb additional water. This additional swelling is opposed by the covalent cross-links, leading to an elastic network retraction force. Thus, the hydrogel will reach an equilibrium swelling level. The additional absorbed water is called ‘free water' or ‘bulk water' and assumed to fill the space between the network chains and/or the centre of larger pores, macro pores, or voids [30]. As shown in Table 3, swelling capabilities of hydrogel are ranged from 169 to 278%, but huge water (670-1113%) can be absorbed by the hydrogels. Hydrogel capability to absorb the oil is an evidence that hydrogel has amphiphilic properties.

## 4. Conclusions

The mixture of carboxymethyl sago or cassava starch and chitosan can be used to produce biohydrogel through chemical cross-linking with methacrylic acid as cross-linking agent. Concentration of cross-linking agent significantly influenced the grafting yield and efficiency and monomer conversion, which is also reflected in the hydrogel swelling and solubility properties and water absorption capacity. The highest grafting yield, grafting efficiency, and monomer conversion were achieved by experiment using 0.550 g of methacrylic acid per g of CMS-chitosan mixture.

## Figures and Tables

**Table 1:** Characteristics of native and carboxymethyl sago and cassava starch.

Characteristics	Sago Starch		Cassava Starch	
	Native	CMS	Native	CMS
Swelling power at 70°C (%)	47.48	137.49	109.50	143.81
Solubility at 70°C (%)	31.83	64.83	26.00	61.18
Water absorption capacity (%)	90.30	789.38	173.00	1022.76
Oil absorption capacity (%)	66.40	148.09	170.00	152.68

**Table 2:** Methacrylic acid grafting parameters on CMS-chitosan*∗*.

Grafting Parameters	Methacrylic Acid Concentration (g/g CMS-chitosan)		
	0.275	0.550	0.825
Biohydrogel: Carboxymethyl sago starch-chitosan			
Grafting Yield (%)	14.85 ± 0.22^a^	46.12 ± 0.91^c^	36.76 ± 0.90^b^
Grafting Efficiency (%)	83.81 ± 1.34^a^	85.62 ± 2.63^ab^	88.37 ± 1.76^b^
Monomer conversion (%)	64.46 ± 0.62^b^	98.04 ± 1.15^c^	50.45 ± 0.27^a^
Biohydrogel: Carboxymethyl cassava starch-chitosan			
Grafting Yield (%)	16.31 ± 0.27^a^	47.09 ± 0.64^c^	37.19 ± 0.30^b^
Grafting Efficiency (%)	84.37 ± 2.35^a^	86.75 ± 1.08^ab^	89.21 ± 0.65^b^
Monomer conversion (%)	70.35 ± 1.16^b^	98.80 ± 0.17^c^	50.57± 0.07^a^

*∗* indicates that means within a row related to particular parameter with the same superscript letter are not significantly different as *α*=0.05 confidence level.

**Table 3:** Characteristics of CMS-chitosan based biohydrogel*∗*.

Characteristics	Methacrylic Acid Concentration (g/g CMS-chitosan)		
	0.275	0.550	0.825
Biohydrogel: Carboxymethyl sago starch-chitosan			
Swelling power (%)	169.52 + 5.13^a^	278.15 + 13.95^c^	197.21 + 11.55^b^
Solubility (%)	59.03 + 1.81^c^	36.03 + 1.77^a^	50.85 + 2.86^b^
Water absorption capacity (%)	781.82 + 13.45^b^	1046.28 + 64.15^c^	669.94 + 40.15^a^
Oil absorption capacity (%)	255.15 + 10.72^a^	250.12 + 6.30^a^	251.27 + 11.64^a^
Biohydrogel: Carboxymethyl cassava starch-chitosan			
Swelling power (%)	198.88 + 4.23^b^	230.92 + 5.64^c^	180.82 + 3.53^a^
Solubility (%)	52.41 + 1.17^b^	43.33 + 1.05^a^	55.32 + 1.10^c^
Water absorption capacity (%)	955.91 + 49.42^b^	1113.26 + 39.11^c^	893.13 + 10.38^a^
Oil absorption capacity (%)	256.84 + 18.91^a^	250.29 + 7.59^a^	253.38 + 7.83^a^

*∗* indicates that means within a column related to particular parameter with the same superscript letter are not significantly different as *α*=0.05 confidence level.

## Data Availability

The data used to support the findings of this study are available in IPB Scientific Repository (https://repository.ipb.ac.id).
